# Enthalpies of Adduct Formation between Boron Trifluoride and Selected Organic Bases in Solution: Toward an Accurate Theoretical Entry to Lewis Basicity

**DOI:** 10.3390/molecules26216659

**Published:** 2021-11-04

**Authors:** Jean-François Gal, Pierre-Charles Maria, Manuel Yáñez, Otilia Mó

**Affiliations:** 1 Institut de Chimie de Nice, UMR 7272, Université Côte d’Azur, Parc Valrose, 06108 Nice, France; Pierre-Charles.MARIA@univ-cotedazur.fr; 2 Departamento de Química (Módulo 13, Facultad de Ciencias) and Institute of Advanced Chemical Sciences (IadChem), Campus de Excelencia UAM-CSIC, Cantoblanco, Universidad Autónoma de Madrid, 28049 Madrid, Spain; manuel.yanez@uam.es (M.Y.); otilia.mo@uam.es (O.M.)

**Keywords:** BF_3_ enthalpies, Lewis basicity, specific solvation, high-level ab initio calculations, dichloromethane, boron trifluoride

## Abstract

The Lewis basicity of selected organic bases, modeled by the enthalpies of adduct formation between gaseous BF_3_ and bases in dichloromethane (DCM) solution, is critically examined. Although experimental enthalpies for a large number of molecules have been reported in the literature, it may be desirable to estimate missing or uncertain data for important Lewis bases. We decided to use high-level ab initio procedures, combined with a polarized continuum solvation model, in which the solvated species were the clusters formed by specific hydrogen bonding of DCM with the Lewis base and the Lewis base/BF_3_ adduct. This mode of interaction with DCM corresponds to a specific solvation model (SSM). The results essentially showed that the enthalpy of BF_3_ adduct formation in DCM solution was clearly influenced by specific interactions, with DCM acting as hydrogen-bonding donor (HBD) molecule in two ways: base/DCM and adduct/DCM, confirming that specific solvation is an important contribution to experimentally determined Lewis basicity scales. This analysis allowed us to conclude that there are reasons to suspect some gas-phase values to be in error by more than the stated experimental uncertainty. Some experimental values in DCM solution that were uncertain for identified reasons could be complemented by the computed values.

## 1. Introduction

The quantitative characterization of the Lewis basicity of a molecule or a functional group in a complex system is frequently a prerequisite for understanding the Lewis acid/base interactions, which are pivotal in many fundamental and applied fields. The IUPAC definition of Lewis basicity as the “thermodynamic tendency of a substance to act as a Lewis base” is fundamentally correct, but, for practical realization, it is primarily measured either as Gibbs energies or enthalpies of adduct formation relative to a standard Lewis acid, in the gas phase or in an inert (or assumed so) solvent [1,2,3,4,5,6,7,8,9].

Basicity is a multidimensional property [10,11,12], but characterization of an interaction with a multiparametric model is rather complex and requires an extended set of carefully chosen data.

For these reasons, the use of unidimensional scales (using a single reference Lewis acid) is often preferred, for example, by looking at a simple linear regression. There are several critically evaluated Lewis basicity scales, covering a respectable range of bases [5]. These scales deal with the most useful interactions, including adduct formation with classical Lewis acids (in particular SbCl_5_ and BF_3_), hydrogen bonding, halogen bonding, and gas-phase metal cation affinities. In fact, one of the most cited Lewis basicity scales is the so-called “Donor Number” (DN) developed by Viktor Gutmann and his followers [13,14]. The DN was initially defined as the calorimetric measurement of the enthalpy of adduct formation between SbCl_5_ and a Lewis base (LB) both dissolved in 1,2-dichloroethane (DCE).

SbCl_5(DCE)_ + LB_(DCE)_ → [LB-SbCl_5_]_(DCE)_ (1)

Despite various drawbacks, in particular experimental problems [5], the use of secondary non-calorimetric measurements to expand the scale, and traceability of the data [15], the DN scale is still cited as a fundamental reference in state-of-the-art research on materials [16,17,18,19,20,21,22,23,24], to cite only a few recent works.

Using a similar calorimetric definition, two of us established a Lewis basicity scale founded on the enthalpies of reaction between gaseous BF_3_ and a Lewis base in dichloromethane (DCM) solution [5,25]:

BF_3(gas)_ + LB_(DCM)_ → [LB-BF_3_]_(DCM)_ (2a)

Measurements were also carried out in nitrobenzene (NB) solution:

BF_3(gas)_ + LB_(NB)_ → [LB-BF_3_]_(NB)_ (2b)

This Δ*H*BF_3_ scale (BF_3_A in [5] where A was used by analogy with PA, the proton affinity measured by an enthalpy) presents several advantages over DN, particularly with regard to the range of enthalpies covered via a single technique, the wide structural variation among the LBs, and its rigorous traceability from the same laboratory, including repeatability tests of calorimetric measurements [26].

In fact, the DN and Δ*H*BF_3_ scales are strongly correlated [5,25], within the range of O- and N-bases for which data are available.

Owing to the need for data on the Lewis basicity of molecules not experimentally characterized [27], several attempts were recently made to estimate either SbCl_5_ or BF_3_ enthalpies of adduct formation by quantum chemistry methods; see, for example, [22,28,29,30].

We also contributed to the evaluation of high-level theoretical methods for calculating the enthalpies for reactions (1) and (2) [31].

There are also data corresponding to BF_3_ enthalpies experimentally determined in the gas phase:

BF_3(gas)_ + LB_(gas)_ → [LB-BF_3_]_(gas)_ (3)

In this work, we investigate the best available ab initio methods compatible with the size of the adducts to calculate BF_3_ enthalpies of adduct formation, i.e., reactions (2) and (3). In the case of reactions (2), we chose a series of representative Lewis bases covering the maximum span of the Δ*H*BF_3_ scale. Examination of reaction (3) was limited to the data available in the literature.

The energetics associated with the interaction of the different bases under study with BF_3_ were investigated using a G4* ab initio method that is described in detail in the Section 3.2. Our aim was to determine how close we could come to the experimental enthalpies of adduct formation, both in the gas phase and in solution. For measurements in DCM solution using Δ*H*BF_3_, for which there is a large choice of experimental data [5], the examination of models of solvent effects was believed to be essential.

From the definitions of DN and Δ*H*BF_3_ following reactions (1) and (2) respectively, the two Lewis basicity scales correspond to Lewis bases and their adducts as solutes diluted in a given solvent (solute scales). This point is often overlooked, and these scales are very frequently treated as solvent scales, leading one to believe that interactions between solvent and solute molecules are only considered in terms of Lewis acid/Lewis base binding [32,33,34]. This approximation may be an acceptable approximation for weakly associated liquids used as solvents, but is certainly not valid for self-associated liquids [35].

## 2. Results and Discussion

### 2.1. Gas Phase Enthalpies

As a prerequisite for potential comparisons between theoretical and experimental enthalpies of BF_3_ adduct formation with bases and adducts in solution (reaction 2), an exploratory analysis of gas-phase data was conducted on seven selected Lewis bases. As the reaction of adduct formation with BF_3_ is always exothermic (negative values), we systematically list and discuss −Δ*H* values for convenience. The enthalpies were calculated at a slightly modified G4 level, described in the Section 3.2, and we label this procedure G4*, to distinguish it from the standard G4 method. The experimental and calculated enthalpies of reaction (3) at the G4* level are listed in Table 1. The B3LYP/aug-cc-pVTZ optimized geometries of the BF_3_ complexes in the gas phase are given in Table S4 of the Supplementary Materials.

The calculated values were in fair agreement for the O-bases, but rather significant deviations were observed for the N-, S- and P-bases. It should be noted that most of these experimental enthalpies were obtained via tensimetric measurements of the dissociation of the gas-phase adducts at different temperatures, which are subject to systematic errors, in particular for the early studies [45].

The gas-phase trimethylamine experimental enthalpy listed in Table 1 was estimated from a thermochemical cycle involving conjectured quantities for both the enthalpy of formation of the solid trimethylamine/boron trifluoride adduct (42 ± 2 kcal·mol^−1^) and for its enthalpy of sublimation (15 kcal·mol^−1^) [38]; a higher reported value (68.9 kJ·mol^−1^ at 393 K) [46] would worsen the deviation between calculated and experimental values. Therefore, the often-quoted value reported in Table 1 appears to be a rough estimate, and the calculated value is probably a better approximation of the true one. As a matter of fact, the G4 calculated proton affinity (PA) of trimethylamine (950.4 kJ·mol^−1^) was in very good agreement with the experimental value (948.9 kJ·mol^−1^) [47], showing that the G4 method reproduced very well the enthalpies of both the neutral trimethylamine and its protonated form, and it is not likely that it overestimated the stability of the trimethylamine/BF_3_ adduct. The good agreement between the experimental PAs and the G4-calculated values was also observed for all the other Lewis bases included in this study, as shown in Table S3 of the Supplementary Materials.

For the tetrahydrothiophene/BF_3_ adduct [44], the authors reported some decomposition during the vaporization of the solid adduct. In this case, the G4 calculated PA (848.7 kJ·mol^−1^) was again in excellent agreement with the experimental value (849.1 kJ·mol^−1^) [47], which leads us to believe that the theoretical estimate for the enthalpy of formation of the tetrahydrothiophene/BF_3_ adduct is probably the closest to the correct value. A similar decomposition problem was reported for the trimethyl phosphine/BF_3_ adduct [43], for which the theoretical estimate, as was the case for trimethylamine, is significantly larger than the experimental value, whose cited source of data [48] is difficult to trace.

To summarize, there are reasons to suspect some gas-phase values to be in error by more than the stated experimental uncertainty.

**Figure 1:**
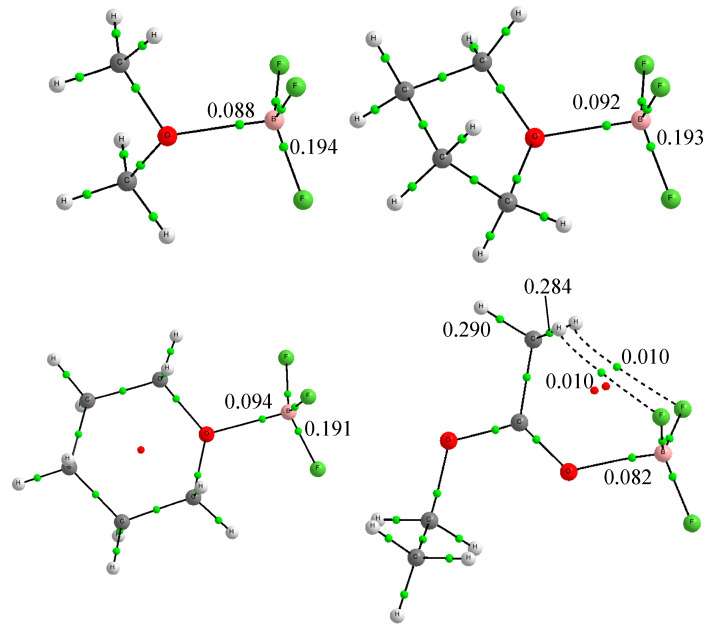
Molecular graphs for the complexes between dimethyl ether, tetrahydrofuran, tetrahydropyran and ethyl acetate with BF_3_. The green dots show the positions of the bond critical points, whose electron density in a.u. is indicated for relevant cases.

It is also interesting to mention that in principle one could naively think that the basicity of the compounds included in our analysis with respect to BF_3_ should follow the same trends as their intrinsic (gas-phase) Brønsted basicity, i.e., the trend exhibited by the gas-phase proton affinities. This was indeed the case when dimethyl ether was compared with tetrahydrofuran and tetrahydropyran. Dimethyl ether exhibited a BF_3_ enthalpy smaller than tetrahydrofuran and tetrahydropyran, as was the case for their intrinsic Brønsted basicities (PAs): 792.0 kJ·mol^−1^ for dimethyl ether and 822.1 and 822.8 kJ·mol^−1^ [47], respectively, for tetrahydrofuran and tetrahydropyran. This result seems to be consistent, as shown in Figure 1, with the electron densities at the corresponding B-O bond critical points (BCPs), being lower for dimethyl ether/BF_3_ complex than for the complexes involving tetrahydrofuran and tetrahydropyran. In addition, consistently, the Wiberg bond order obtained in the framework of the NBO approach for the B-O interaction was also smaller for the dimethyl ether/BF_3_ complex (0.377) than for the tetrahydrofuran/BF_3_ and tetrahydropyran/BF_3_ ones (0.386 and 0.398, respectively). However, when considering the couple tetrahydrofuran and tetrahydropyran, we observed that whereas their PAs were practically equal, as mentioned above, the BF_3_ affinity of tetrahydrofuran was clearly larger (both the theoretical estimates and the experimental measurements) than that of tetrahydropyran. More significant are the changes when looking at the values associated with ethyl acetate. Both the calculated and the experimental BF_3_ affinities for ethyl acetate were the lowest of the four oxygen bases included in Table 1, whereas its proton affinity (835.7 kJ·mol^−1^) was the largest of the series. These results are in line with those reported by A. Rauk et al. [49] based on MP2 ab initio calculations.

It must be ratified, then, that no correlation should be expected between BF_3_ interaction enthalpies and proton affinities. As a matter of fact, protonation is a very different process in which the attachment of a proton to the basic site of the base implies a huge charge transfer from the base, with a concomitant strong polarization of its charge density, to the incoming proton, resulting in the formation of a new covalent bond between the basic site and the proton. On top of that, another important difference between the protonation process and the formation of BF_3_ complexes is the possibility, in the last case, of additional non-covalent interactions between the fluorine atoms of BF_3_ and other atoms of the base, which are not possible in protonation processes. This was quite evident when looking at the molecular graph of the ethyl acetate/BF_3_ complex, which shows the formation of C-H···F hydrogen bonds between the C-H bonds of the base and the BF_3_ fluorine atoms. The formation of these hydrogen bonds was also corroborated by the NBO analysis, which shows a non-negligible population of the corresponding C-H antibonding orbitals, resulting in a lengthening of 0.006 Å (1 Å = 100 pm) in the two C-H bonds involved, and a decrease in the electron density at the C-H BCP (see Figure 1).

### 2.2. Enthalpies in Solution

From the very large Δ*H*BF_3_ data set measured in DCM [5,10,25,31], we selected a series of Lewis bases which partly overlap the experimental gas-phase enthalpies of reaction (3) and cover the largest possible range of Lewis basicities, with a wide variation among functional groups. The selection included quinuclidine, one of the strongest bases (150 kJ·mol^−1^) in this solute scale, and nitrobenzene (acting as a Lewis base, 36 kJ·mol^−1^) as the weakest.

Some BF_3_ enthalpies were also measured in nitrobenzene as solvent [25], not only to check the medium effect, but also due to solubility problems or because secondary reactions between DCM and some bases or their adducts plagued calorimetric measurements. The experimental data are listed in Table 2. The listed uncertainties, defined as the 95% confidence interval, correspond to the short-term repeatability. Systematic errors, discussed in detail in Section 3, may add approximately 1 to 2 kJ·mol^−1^ to the listed uncertainties.

Several experimental data were unpublished, so the experimental method [26] is briefly recalled in the Section 3.

For a series of 12 well-behaved Lewis bases, a linear least square regression between the BF_3_ enthalpies in the two solvents (dichloromethane and nitrobenzene) leads to Equation (4) (coefficient of determination *R*^2^ = 0.9940; units kJ·mol^−1^) [25].

−Δ*H*_(DCM solution)_ = 0.958 [−Δ*H*_(NB solution)_] − 0.31 (4)

As explained in Section 3, amines may be subject to secondary reactions in DCM. This is the origin of the higher-than-usual uncertainty of the enthalpy value for quinuclidine in DCM. For this reason, the enthalpy value was estimated using Equation (4) as well. This equation was also applied to the enthalpy of solution of BF_3_ in nitrobenzene, considered as a Lewis base in nitrobenzene solution, leading to an estimation of the enthalpy in DCM.

**Figure 2:**
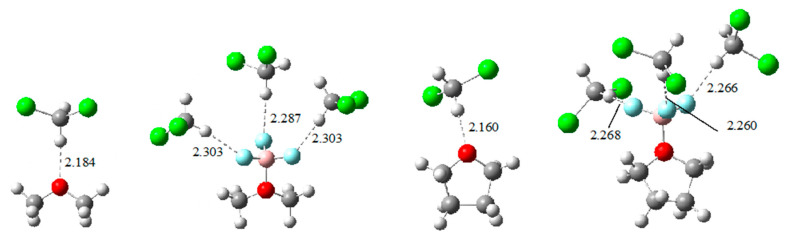
Examples of B3LYP/6.31+G(d,p) optimized structures of hydrogen bonded species which were considered for the specific solvation effect by DCM for dimethyl ether and tetrahydrofuran as suitable example. Before adduct formation, the Lewis base is in interaction with one DCM molecule. Meanwhile, the BF_3_ adduct is in interaction with three DCM molecules through the three fluorine atoms. H-Bond distances in Å.

Table 2 also gives the ab initio calculated enthalpies in DCM and NB solution, and in the gas phase for comparison. Our objective was to reproduce as accurately as possible the absolute enthalpies of BF_3_ adduct formation in solution, via reactions (2). Previously, we applied the polarized continuum model (PCM) to calculated enthalpy values, using the standard G4 method, for 8 phosphoryl bases [31], and compared with experimentally available data. Although the results were promising, the enthalpies calculated in the two solvents were quite close, with maximum differences of about 2 kJ·mol^−1^ for values in the range 27 to 129 kJ·mol^−1^. This result was not completely consistent with Equation (4), which indicates that enthalpies in DCM should be approximately 96% of those in NB. The continuous solvation model for DCM was applied to 12 Lewis bases pertaining to the present study, and the results are given in Table S1 of the Supplementary Materials. The calculated values were almost always too high as compared to the experimental ones. A part of this discrepancy was attributed to a specific solvent effect of DCM, which is known to be a weak hydrogen bond donor (HBD) [50,51,52,53,54]. It may be considered that the interactions of DCM with the bases and BF_3_ adducts were primarily dipole/dipole, but the structure of the interacting species (Figure 2) indicates an enhancement by directional hydrogen bonding, through the appropriate orientation of the DCM molecule.

It may be argued that halogen bonding may be additionally involved in DCM/Lewis base interaction, but solid phase studies show that hydrogen bonding is dominant for F, O, N, S and P-bases, while it competes with halogen bonding for Cl, Br, or I-bases [55]. Therefore, we can conclude that in our case, H-bonding was the most significant non-specific solvation in DCM solution.

The enthalpy of BF_3_ adduct formation may be influenced by specific interactions with DCM acting as HBD molecule in two ways: base/DCM and adduct/DCM. Reactions (2) corresponds to the addition of gaseous BF_3_ to a Lewis base solution, so H-bonding to the isolated BF_3_ is not relevant here. The specific solvation of Lewis bases by DCM was suggested by Drago and coworkers [51], and a correction to the BF_3_ enthalpies for DCM solvation was proposed [52]. Although several modes of H-bonding may be expected for the interaction Lewis base/DCM or BF_3_ adduct/DCM, we opted for a simple model: one H-bond with the electron lone pair of the Lewis base, and three H-bonds (one on each fluorine of coordinated BF_3_) for the adduct. Figure 2 shows two typical cases of calculated structures of the solutes in reaction (2a) interacting via H-bonding with DCM. In addition to the specific solvation characterized by the Lewis base/DCM and by the adduct/DCM interactions, the PCM model was applied to these H-bonded systems to allow for the non-specific solvation.

**Figure 3:**
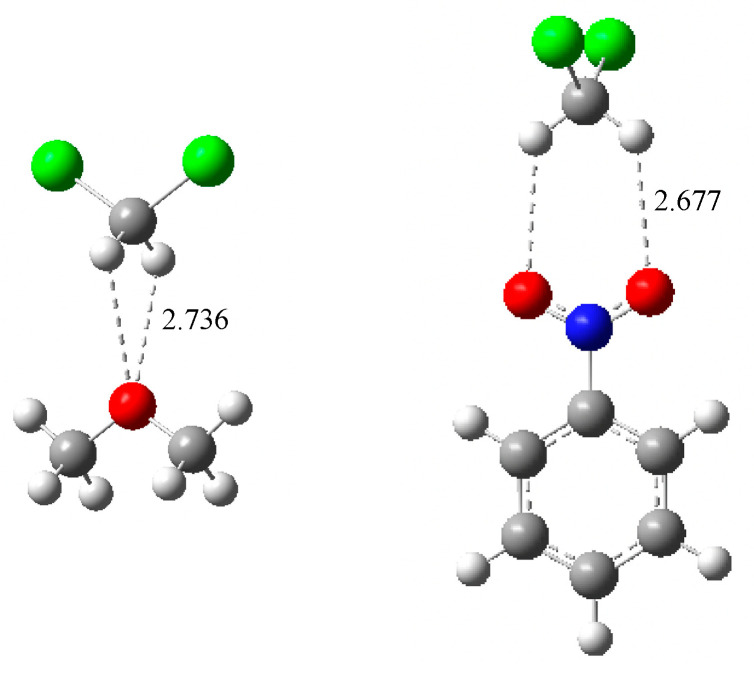
Stable structures for the solvation complexes between dimethyl ether and nitrobenzene with DCM stabilized by two H-bonds. None of them are local minima of the potential energy surface, but rather saddle points with two imaginary frequencies. Hydrogen bond distances in Å.

Other arrangements with two H-bonds between the basic site and the DCM molecule were higher in energy, and frequently we did not find minima but rather saddle points. Two paradigmatic examples are shown in Figure 3.

Solvation by nitrobenzene was considered essentially due to non-specific interaction, and the PCM model was directly applied. In Table 2, we can see that most calculated values at the G4*/specific solvation model (SSM) were within ±6 kJ·mol^−1^ (mean unsigned deviation = 4.2 kJ·mol^−1^) of the experimental values for reaction (2a). There were two exceptions to this fit: quinuclidine and HMPA. Quinuclidine presents some experimental problems for measurements in DCM, explaining the larger-than-usual uncertainty. For this reason, its enthalpy was also measured in NB, and an estimation of the enthalpy in DCM was obtained using Equation (4), showing a better agreement with the calculated values, although still differing by 9.5 kJ·mol^−1^. The second exception was HMPA. Our simplified SSM considered only one H-bond on the basic center of the Lewis base, the phosphoryl oxygen atom, but the three nitrogen atoms of the dimethylamino groups are also potential basic sites, although weaker than the phosphoryl. The cluster corresponding to multiple DCM molecules bound to HMPA is not amenable to G4 calculations and the hypothesis of an additional specific solvation on the nitrogen sites of HMPA could not be tested. Overall, the SSM + PCM solvation scheme appeared to perform satisfactorily for reproducing the enthalpies of adduct formation with BF_3_ for the N-, O-, P- and S-bases studied.

The success of PCM solvation applied to the measurements conducted in nitrobenzene as solvent was mitigated. The calculated −Δ*H* values for the six cases in Table 2 were almost all too large, with disagreements with experimental enthalpies up to 12 kJ·mol^−1^. It should be noted, as far as the calculations are concerned, that our theoretical treatment included geometry optimization of the complex when solvent effects were accounted for. Curiously, for HPMA, these values were in worse agreement with the experimental ones than those obtained when no geometry optimization of the complex was carried out. Provisionally, we assigned this observation to the neglect of the potential solvation of the NMe_2_ groups of HMPA in the SSM.

We wish to point out that the high computational level required to reproduce Δ*H*BF_3_ values is not attainable for DN (i.e., enthalpies of adduct formation with SbCl_5_ in solution). This is a further advantage of using Δ*H*BF_3_ as a Lewis basicity scale, as it widens the possibilities of evaluating this type of basicity for species other than those already experimentally characterized by calorimetry [5].

**Figure 4:**
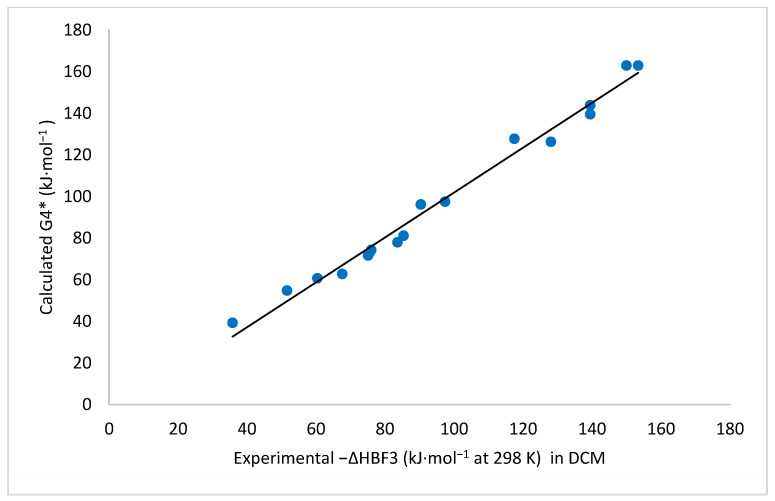
Linear correlation between the experimental enthalpies of adduct formation (−Δ*H*BF_3exp_._DCM_) and the G4* calculated values obtained using the discrete solvation model.

Finally, it is worth mentioning that, as illustrated in Figure 4, there was a rather good linear correlation between the experimental enthalpies Δ*H*BF_3_ in DCM solution and the G4* calculated values obtained using the discrete solvation model (SSM). This linear correlation obeys the equation:

−Δ*H*BF_3calc_ = 1.078 (−Δ*H*BF_3exp_._DCM_) − 5.900; *R*^2^ = 0.985 (5)

## 3. Materials and Methods

### 3.1. Calorimetric Method

We succinctly recall the essentials of the calorimetric method utilized for our enthalpy measurements [26]. The calorimeter was a Tian-Calvet differential microcalorimeter, with two 17 mm diameter cells. The measuring and reference cells were made of borosilicate glass. All measurements were taken at 25.0 ± 0.1 °C, under ambient atmospheric pressure. The system was calibrated using the Joule effect, giving uncertainties on the calorimeter calibration constant much less than 0.1% (short time precision at the 95% confidence level for a series of more than 10 measurements), and approximately 0.5% for long-term accuracy (drift of the constant over 5 years). The BF_3_ quantity injected into the Lewis base solution was measured by a mercury manometer (PVT measurements) in a constant temperature room at 20.0 ± 0.1 °C. The repeatability of the enthalpies reported in Table 2 (usual range 0.5–1% for approximately 10 consecutive measurements on the same solution) was largely due to errors in the BF_3_ measurements. The long-term reproducibility (several years) of the calibration constant was better than 0.5% when considering the drift of the calibration constant. Systematic errors also arose from measurements of gas quantities, which were evaluated as approximately 0.2% [26]. Our calorimetric measurements gave absolute enthalpies of reaction measured consistently using the same protocol. Comparing our data to those measured by H. C. Brown and coworkers for five Lewis bases in NB solution, using a different calorimetric procedure, we observed fair to excellent agreement [25], showing that systematic errors on our enthalpies were less than 1%.

In some cases, we had to consider larger than usual errors in the heat effect, because of secondary reactions. The amines tend to react with methylene chloride to very different degrees [51,56]. For trimethylamine and *N*-methylpyrrolidine, we did not observe significant DCM/amine or DCM/adduct reactions, but quinuclidine displayed this problem. This DCM/quinuclidine concentration-dependent reaction was previously observed by Drago et al. [51]. The larger uncertainty on the enthalpy of adduct formation in DCM for this compound was imputed to such problem.

### 3.2. Computational

The G4* approach used in our calculations is a slight modification of the G4 ab initio method [57]. The G4 theory is a composite formalism based on the use of Møller–Plesset perturbation theory up to fourth order and CCSD(T) coupled cluster theory to accurately describe electron correlation effects. The method includes a final correction for the Hartree–Fock limit, evaluated using an extrapolation procedure and quadruple-zeta and quintuple-zeta basis sets. It must be mentioned, however, that in this study we introduced a slight modification as far as the optimized geometries are concerned. Indeed, since in our case we were dealing with rather weak interactions which usually required the use of diffuse functions which are not included in the standard G4 formalism, which uses B3LYP/6-31G(2df,p) optimized geometries, we decided to use B3LYP/aug-cc-pVTZ optimized geometries (see Table S4 of the Supplementary Materials) instead. These slightly modified G4 calculations are named as G4* elsewhere.

To analyze the bonding characteristics of some of the complexes under investigation chosen as suitable illustrations, we used two different approaches, namely the atoms in molecules (AIM) method [58] and the Natural Bond Orbital (NBO) approach [59].

The AIM method permits the location of the so-called bond critical points (BCPs) and calculation of the corresponding electron density, whose value at the BCPs is a good measure of the strength of the linkage as well as providing information about its covalent character, through the values and signs of the Laplacian and energy density. The NBO method provides, through an appropriate molecular orbital localization scheme, a description of the systems in terms of a Lewis type representation. When dealing with intermolecular interactions, this approach is very well suited to estimating the relative strength of the interaction between the groups involved.

To account for solvation effects, we used semi-continuum or cluster-continuum approaches [60]. Often used in the context of solvation of ions by water, these approaches are supposed to complete the electrostatic description of the polarized continuum model (PCM) developed by Tomasi et al. [61]. In the model, the continuum approaches are used on clusters in which the molecule and its adduct with BF_3_ are specifically solvated by one and three molecules of DCM, respectively. This notably increases the size of the systems to be investigated, and therefore these calculations to account for the solvation stabilization effects were carried out at the B3LYP/6-31+G(d,p) level of theory.

## 4. Conclusions

The experimental enthalpies of adduct formation between neutral Lewis bases and boron trifluoride measured in dichloromethane are available for about 350 molecules [5]. One of our objectives was the reproduction of absolute enthalpy values using state-of-the-art ab initio approaches. A previous work on a series of phosphoryl compounds using G4 calculations and a continuous solvation model was rather successful, but application to the various bases included in the present work led us to conclude that the solvation model should be refined. An improved G4* combined with a solvation model including specific hydrogen bonding to the Lewis base and the BF_3_ adduct, completed by a continuous solvation model (PCM), was more satisfactory for the reproduction of the experimental enthalpies in DCM.

Comparison of BF_3_ enthalpies measured and calculated in the gas-phase and in DCM solution showed that the solvent effect was sizeable. The use of DCM for reaction (2a), similarly to the use of DCE (1,2-dichloroethane) for reaction (1), was dictated by solubility problems, mainly for the BF_3_ adducts, which are poorly soluble in low polarity solvents such as tetrachloromethane and alkanes.

Solvation is an important component of Lewis basicity measurements. A recent computational study focused on Gibbs energies of adduct formation between antimony pentahalides and group 13 Lewis acids (among them SbCl_5_ and BF_3_), and the Lewis bases acetonitrile and pyridine, examined the solvent effect on the basis of a continuous dielectric model. The authors concluded that electrostatic, dispersion and electron-repulsive solute–solvent interactions were essential for the prediction of solvation effects [62], while the solvents selected for the study did not include possible hydrogen bond-donor solvents. It is worth mentioning that G4/G4* calculations are not currently possible for antimony pentahalide adducts, in particular for estimating DN via reaction (1).

Our hybrid discrete-continuum solvation model approach appeared to be efficient for the selected Lewis bases. Most experimental Δ*H*BF_3_ were reproduced within ±6 kJ mol^−1^, opening the way to the theoretical evaluation of Lewis basicity, as defined by reaction (2a), in particular for molecules not experimentally characterized. At the cost of time-consuming and expensive computations, more sophisticated DCM/Lewis bases and DCM/BF_3_ adducts may be devised, but for more widespread applicability, it is probably advisable to search for less expensive methods.

The importance of solvent effects on Lewis basicity points out the necessity of distinguishing between the Lewis basicity defined by reactions (1) and (2) and “solvent basicity”. Solvent basicity is often an ill-defined property, considering only the experimental or computed thermochemistry of reactions (1), (2a), (2b) and (3), and ignoring the “bulk effect”, i.e., the variable solvation of each solvent [35]. Further experimental and computational studies are planned on the Lewis basicity of solvents as opposed to solutes, i.e., the enthalpy of solution of BF_3_ in bulk liquids.

## Figures and Tables

**Table 1:** Gas-phase enthalpies (−Δ*H*) of adduct formation with Lewis bases; values in kJ·mol^−1^ at 298 K unless otherwise noted.

Lewis Base	G4*-Calculated *^a^*	Experimental	Δ(Calc. − Exp.)
Trimethylamine	126.4	111.3 ± 8.4 *^b^* [36,37,38]	15.1
Dimethyl ether	62.4	57.1 ± 0.8 [39]	5.3
Tetrahydrofuran	72.5	70.3 ± 0.8 [40]	2.2
Tetrahydropyran	69.0	64.5 ± 0.8 [40]	4.5
Ethyl acetate	55.9	53.6 ± 2.9 [41]	2.3
Trimethylphosphine	66.1	79.1 [42,43]	−13.0
Tetrahydrothiophene	37.9	21.8 ± 1.7 [44]	16.1

*^a^* see text and Section 3.2;*^b^* at 273 K.

**Table 2:** Experimental and calculated enthalpies of adduct formation (−Δ*H*, kJ·mol^−1^ at 298 K)) between BF_3_ and Lewis bases in dichloromethane (DCM) and nitrobenzene (NB) solutions (reaction (2a) and (2b)); kJ·mol^−1^). The signification of uncertainties on experimental enthalpies is discussed in the text. G4*-calculated gas-phase enthalpies are listed in the last column.

	Solvent CH_2_Cl_2_ (DCM)			Solvent PhNO_2_ (NB)			Gas Phase
Lewis Base	Experimental in DCM *^a^*	G4* + Discrete Solvation Model *^b^*	Δ = Calc − Exp	Experimental in NB *^a^*	G4* + Continuous Solvation Model *^c^*	Δ = Calc − Exp	G4*
Trimethylamine	139.5 ± 1.8	145.7	6.2				126.4*^e^*
*N*-Methylpyrrolidine	139.5 ± 0.8	143.8	4.3				125.2
Quinuclidine	150.01 ± 3.48 [153.4 ± 0.9] *^d^*	162.9	12.9 [9.5]	160.5 ± 0.9	171.2	10.7	139.1
Pyridine	128.1 ± 0.5	126.3	−1.8	137.9 ± 0.7	137.4	−0.5	100.4*^e^*
Acetonitrile	60.4 ±0.5	60.7	−0.3				32.3
Dimethyl ether	83.6 ± 0.2	78.0	−5.6				62.4
Tetrahydrofuran	90.4 ± 0.3	96.2	5.8	93.0 ± 0.3	103.9	10.9	74.8
Tetrahydropyran	85.4 ± 0.5	81.2	−4.2				69.0*^e^*
Acetone	76.0 ± 0.2	74.3	−1.7	78.1 ± 0.3	82.7	4.6	54.2
Ethyl acetate	75.6 ± 0.3	73.2	−2.4				55.9
γ-Butyrolactone	75.1 ± 1.2	71.7	−3.4				53.1
Dimethyl carbonate	67.6 ± 0.4	62.8	−4.8				30.8
Nitrobenzene	[35.8 ± 1.4] *^d,f^*	39.3	[3.5]	37.7 ± 1.4 *^g^*	45.3	7.6	21.0*^e^*
Hexamethyl-phosphoramide (HMPA)	117.5 ± 0.5	127.7	10.2	123.1 ± 0.5	135.2 (121.9) *^i^*	12.1 (−1.2)	101.3 *^h^*
Trimethylphosphine	97.4 ± 0.3	97.5	0.1				66.1
Tetrahydrothiophene	51.6 ± 0.2	54.8	3.2				37.9

*^a^* Experimental values corresponding to the reaction: BF_3(gas)_ + LB_(solution)_ → [LB-BF_3_]_(solution)_. *^b^* Solvation effects are calculated using a model combining specific interactions (SSM) and a continuum model; see text. *^c^* Solvation effects are calculated using a continuum model, including geometry optimization at the B3LYP/6-31+g(d,p) level of theory. *^d^* Secondary value calculated from measurements in NB [25]. The assigned uncertainty corresponds to the repeatability, see text, but additional uncertainties are expected by converting to a value in DCM as solvent. *^e^* In these particular cases, the standard G4 yields a higher value than the one obtained with the non-standard G4* procedure; see Table S2. *^f^* Value for the Lewis base PhNO_2_ in CH_2_Cl_2_ solution, estimated using Equation (4), see text. *^g^* Value measured for the dissolution of BF_3_ in pure PhNO_2_, corresponding to the adduct formation with the Lewis base PhNO_2_ in PhNO_2_ solution. *^h^* Published value obtained by extrapolation of the G4MP2 results [31]. *^i^* Solvation effects do not include geometry optimization. The previously reported value (119.3 kJ·mol^−1^) [31] is very close but not strictly equal because the basis set used in the calculation of the solvation effects was different from the one used here.

## Data Availability

Not applicable.

## Supplementary Materials

The following are available online. Table S1. Calculated solvation effects on the enthalpies of BF_3_ adduct formation; Table S2. Comparison of calculated gas-phase enthalpies: G4* vs. standard G4; Table S3. Comparison of experimental gas-phase PAs with G4-calculated values; Table S4. Optimized structures of the adducts formed between boron trifluoride and Lewis bases included in this study.

## Sample Availability

Samples of the compounds not are available from the authors.
