# Supplementary material for: Enthalpies of Adduct Formation between Boron Trifluoride and Selected Organic Bases in Solution: Toward an Accurate Theoretical Entry to Lewis Basicity
Source: Molecules. 2021 Nov 4;26(21):6659. doi: 10.3390/molecules26216659 (PMC8587173; doi:10.3390/molecules26216659)
Supplement: Supplementary file 1 [file molecules-26-06659-s001.zip › molecules-1425970-supplementary.pdf]

Supporting Information

Enthalpies of adduct formation between boron trifluoride and selected organic bases in solution: Toward an accurate theoretical entry to Lewis basicity

Jean-François Gal,[1]* Pierre-Charles Maria,[1] Manuel Yáñez,[2] Otilia Mó,[2]

[1] Institut de Chimie de Nice, UMR 7272, Université Côte d'Azur, Parc Valrose, 06108 NICE, France.

[2] Departamento de Química (Módulo 13, Facultad de Ciencias) and Institute of Advanced Chemical Sciences (IadChem), Universidad Autónoma de Madrid, Campus de Excelencia UAM-CSIC, Cantoblanco, 28049-Madrid, Spain.

Contents

S1. Calculated solvation effects on the enthalpies of BF_3 adduct formation.

S2. Comparison of calculated gas-phase enthalpies: G4* vs. standard G4.

S3. Comparison of experimental gas-phase PAs with G4-calculated values.

S4. Optimized structures of the adducts formed between boron trifluoride and the Lewis bases included in this study.

S1. Calculated solvation effects on the enthalpies of BF_3 adduct formation.

Table S1 compares the results of the polarized continuum solvation model (PCM) and of the specific solvation model (SSM) described in the main text. In summary, the SSM considers the H-bonding specific solvation of the solutes (Lewis base and BF3 adduct) by dichloromethane (DCM), with the non-specific solvation (PCM) applied to the H-bonded systems. The later model gives in general a better agreement with the experimental data. Comparison with calculated gas-phase values shows the significant effect of solvation on the enthalpies of BF_3 adduct formation.

Table S1. Calculation of the DCM solvation effects on the enthalpies of BF_3 adduct formation ($-\Delta H$ in kJ mol^{-1}) by the G4* method (see main text for definition), and comparison with enthalpies measured in DCM; calculated values correspond to optimized geometries.

Lewis base (LB)	Calculated Gas-phase (G4* or G4) [a]	Calculated + Continuous solvation model [b]	Calculated + Specific solvation model [c]	Experimental [d]
Trimethylamine	126.4	154.4	145.7	139.5 ± 1.8
Pyridine	100.4	133.3	126.3	128.1 ± 0.5
Acetonitrile	32.3	64.0	60.7	60.4 ± 0.5
Dimethyl ether	62.4	87.1	78.0	83.6 ± 0.2
Tetrahydrofuran	74.8	97.7	96.2	90.4 ± 0.3
Tetrahydropyran	69.0	91.6	81.2	85.4 ± 0.5
Acetone	54.2	79.2	74.3	76.0 ± 0.2
Ethyl acetate	55.9	78.2	73.2	75.6 ± 0.3
γ-Butyrolactone	53.1	75.4	71.7	75.1 ± 1.2
Dimethyl carbonate	30.8	64.5	62.8	67.6 ± 0.4
Hexamethyl-phosphoramide (HMPA)	101.3 [e]	130.9	127.7	117.5 ± 0.5
Tetrahydrothiophene	37.9	55.9	54.8	51.6 ± 0.2

[a] The standard G4 value is reported when the adduct is calculated to be more stable than with G4*, see Table S2.
[b] Solvation effects are calculated using a polarized continuum model (PCM), including geometry optimization at the B3LYP/6-31+g(d,p) level of theory. Differences between these values and the experimental values lead to a mean unsigned deviation of 5.6 kJ mol^{-1}.
[c] Solvation effects are calculated using a model combing specific interactions and the PCM, including geometry optimization at the B3LYP/6-31+g(d,p) level of theory; see text. Differences between these values and the experimental values lead to a mean unsigned deviation of 4.1 kJ mol^{-1}.
[d] Experimental values corresponding to the reaction: $BF_{3(gas)} + LB_{(DCM)} \rightarrow [LB\text{-}BF_3]_{(DCM)}$
[e] Value published previously, obtained by extrapolation of the G4MP2 results.[1]

[1] Gal, J.-F.; Maria, P.-C.; Yáñez, M.; Mó, O. On the Lewis basicity of phosphoramides: A critical examination of their Donor Number through the comparison of enthalpies of adduct formation with $SbCl_5$ and BF_3. *ChemPhysChem* **2019**, *20*, 2566-2576.

S2. Comparison of calculated gas-phase enthalpies: G4* vs. standard G4.

Table S2. Comparison of calculated gas-phase enthalpies of BF_3 adduct formation by the G4* and the standard G4 methods (kJ mol^{-1} at 298 K). In some cases, the standard G4 yields a higher value (in bold) than the one obtained with G4*. Because higher values reflect most stable adducts, these values were selected for Table 2 in the main text.

Lewis base	G4*	Standard G4
Trimethylamine	125.5	**126.4** [a]
N-Methylpyrrolidine	125.2	124.1
Quinuclidine	139.1	136.6
Pyridine	100.1	**100.4** [a]
Acetonitrile	32.3	25.7
Dimethyl ether	62.4	60.2
Tetrahydrofuran	74.8	72.5
Tetrahydropyran	67.3	**69.0** [a]
Acetone	54.2	50.3
Ethyl acetate	55.9	52.1
γ-Butyrolactone	53.1	52.7
Dimethyl carbonate	30.8	28.6
Nitrobenzene	19.8	**21.0** [a]
Hexamethyl-phosphoramide (HMPA)	(101.3) [b]	
Trimethylphosphine	66.1	59.8
Tetrahydrothiophene	37.9	32.2

[a] The standard G4 yields a higher value than the one obtained with the non-standard procedure.
[b] See footnote *e* of Table S1.

S3. Comparison of experimental gas-phase PAs with G4-calculated values.

Table S3. Comparison of experimental gas-phase proton affinities (PA(exp.)) with calculated G4-values (PA(calc.)) (kJ mol^{-1} at 298 K).

Lewis base	PA (exp.) [a]	PA (calc.)
Trimethylamine	948.9	950.4
N-Methylpyrrolidine	965.6	963.2
Quinuclidine	983.3	981.8
Pyridine	930.0	931.1
Acetonitrile	779.2	774.2
Dimethyl ether	792.0	792.2
Tetrahydrofuran	822.1	828.2
Tetrahydropyran	822.8	827.8
Acetone	812.0	814.8
Ethyl acetate	835.7	830.5
γ-Butyrolactone	840.0	839.5
Dimethyl carbonate	830.2	830.0
Nitrobenzene	800.3	798.5
Hexamethyl-phosphoramide (HMPA)	958.6	964.2 [b]
Trimethylphosphine	958.8	954.7
Tetrahydrothiophene	849.1	848.7

[a] Values taken from NIST Chemistry WebBook, NIST Standard Reference Database No. 69; Linstrom, P.J.; Mallard, W.G. (Eds.) National Institute of Standards and Technology: Gaithersburg, MD, USA, 2014. Available online: http://webbook.nist.gov/chemistry (accessed on 14 September 2021).
[b] Value calculated at the G4MP2 level of theory.

S4. Optimized structures of the adducts formed between boron trifluoride and the Lewis bases included in this study.

Table S4. Cartesian coordinates in Å (1 Å = 100 pm) corresponding to the B3LYP/aug-cc-pVTZ optimized geometries of the adducts formed between boron trifluoride and the different Lewis bases included in this study.

Trimethylamine<br>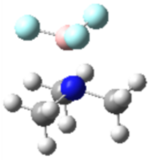	B 0.93515900 -0.00005100 -0.00005700<br>F 1.30240800 -1.27741800 0.36691500<br>F 1.30239100 0.32091800 -1.28976000<br>F 1.30234200 0.95645500 0.92275400<br>N -0.75303300 -0.00001900 -0.00001200<br>C -1.24265800 -0.33907900 1.36305000<br>H -0.86680300 -1.31983600 1.63816400<br>H -0.86606700 0.39795200 2.06576200<br>H -2.33300600 -0.34135100 1.37399200<br>C -1.24293900 -1.01083000 -0.97513200<br>H -0.86681600 -0.75908000 -1.96205200<br>H -0.86693200 -1.98809500 -0.68803000<br>H -2.33329100 -1.01870400 -0.98283100<br>C -1.24270700 1.34999900 -0.38777700<br>H -0.86602200 2.07875900 0.32346100<br>H -0.86702300 1.58973400 -1.37784300<br>H -2.33305500 1.36087600 -0.39028500
N-Methylpyrrolidine<br>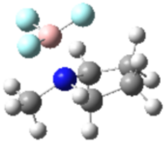	C -0.97975800 0.51287700 1.18400500<br>C -0.97980500 0.51288500 -1.18396100<br>C -2.03506000 -0.51943500 -0.77501400<br>C -2.03503600 -0.51943300 0.77509500<br>H -0.38938500 0.23033400 2.05083500<br>H -1.43612700 1.48586200 1.37402000<br>H -1.43618700 1.48587000 -1.37394900<br>H -0.38946600 0.23035200 -2.05081700<br>H -3.00629300 -0.25052300 -1.18678400<br>H -1.77330000 -1.50374500 -1.15122200<br>H -3.00625400 -0.25051000 1.18689500<br>H -1.77327300 -1.50374400 1.15129700<br>N -0.06609400 0.66424300 0.00000400<br>C 0.60106300 1.99195700 -0.00000700<br>H 1.22587300 2.07429400 0.88419300<br>H 1.22584100 2.07429500 -0.88423000<br>H -0.15139100 2.78057200 0.00000700<br>B 1.14201900 -0.51207900 -0.00002500<br>F 0.51239800 -1.73920200 -0.00005300<br>F 1.86791800 -0.28684100 1.15215600<br>F 1.86791600 -0.28678700 -1.15219700

Quinuclidine<br>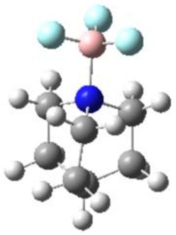	C -0.33542700 -1.11132100 -0.87129400<br>C -1.87356700 -1.21438500 -0.77187600<br>C -2.40567700 0.00009600 0.00010600<br>C -1.87323200 1.27604800 -0.66527100<br>C -0.33496500 1.30973100 -0.52729600<br>H -3.49524400 0.00034100 0.00025900<br>H -2.30994400 -1.25106900 -1.77036100<br>H -2.16374700 -2.13491500 -0.26343600<br>H -0.00136700 -0.89105800 -1.88150900<br>H -2.16415600 1.29681800 -1.71644300<br>H -2.30910700 2.15871900 -0.19665000<br>H 0.00030000 2.07456900 0.16784100<br>H 0.16281300 1.48558100 -1.47663500<br>H 0.16299100 -2.02150400 -0.55008700<br>N 0.17855500 -0.00026900 -0.00021900<br>C -0.33498300 -0.19884800 1.39769400<br>C -1.87311200 -0.06125300 1.43775900<br>H -0.00067700 -1.18372100 1.71211900<br>H 0.16344700 0.53464500 2.02502700<br>H -2.30929700 -0.90757300 1.96894400<br>H -2.16308000 0.83952300 1.98047900<br>B 1.84955200 -0.00004800 -0.00005700<br>F 2.22628500 1.07080700 0.78723100<br>F 2.22650100 -1.21708000 0.53392700<br>F 2.22668200 0.14642300 -1.32078800
Pyridine<br>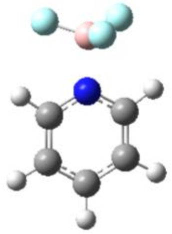	C 0.73149000 -1.16151500 -0.02600600<br>C 2.11641600 -1.20024400 0.00492200<br>C 2.82075800 0.00012200 0.02305600<br>C 2.11619000 1.20036700 0.00491000<br>C 0.73126600 1.16137800 -0.02605300<br>N 0.06418000 -0.00013800 -0.03112300<br>H 3.90505500 0.00021600 0.04669600<br>H 0.11191800 -2.04846400 -0.05618800<br>H 2.62689000 -2.15525500 0.00936100<br>H 2.62649200 2.15546600 0.00936300<br>H 0.11157800 2.04823700 -0.05625000<br>B -1.61804100 -0.00003500 0.00609900<br>F -1.96377500 -1.15064900 -0.64450400<br>F -1.94335900 0.00077400 1.32909900<br>F -1.96372200 1.14990700 -0.64577100
Acetonitrile<br>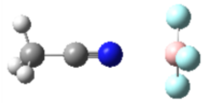	C 1.95967900 -0.00012500 0.00019000<br>N 0.81418500 -0.00016400 0.00030800<br>C 3.41170600 0.00001300 -0.00005900<br>H 3.78317800 0.19129300 1.00577200<br>H 3.78306500 -0.96670900 -0.33743300<br>H 3.78286300 0.77554300 -0.66869400

	B -1.42557600 0.00003200 -0.00005100<br>F -1.56145500 -0.48201000 1.22984100<br>F -1.56097300 -0.82406500 -1.03253000<br>F -1.56077500 1.30624500 -0.19757000
Dimethyl ether<br>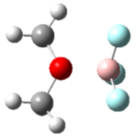	B -0.85102600 0.00001000 0.01891300<br>F -1.27719000 1.15122500 -0.56062300<br>F -1.27724800 -1.15106100 -0.56087100<br>F -0.86831200 -0.00013700 1.38348000<br>O 0.81732100 0.00000600 -0.35495500<br>C 1.52958600 -1.20578700 -0.00360700<br>H 0.92491600 -2.03661200 -0.34748700<br>H 2.49059900 -1.19584500 -0.51384700<br>H 1.66733200 -1.25451400 1.07659000<br>C 1.52964600 1.20575000 -0.00361100<br>H 1.66745200 1.25445200 1.07658100<br>H 2.49063600 1.19579100 -0.51389500<br>H 0.92499200 2.03661200 -0.34743600
Tetrahydrofuran<br>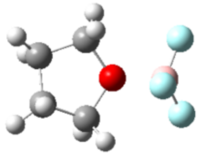	C -0.93817400 -1.19355100 -0.29565900<br>O -0.15085200 0.04276500 -0.43393400<br>C -0.94849900 1.20806700 -0.03561200<br>C -2.38010300 0.71181000 -0.11583600<br>C -2.25367000 -0.75102800 0.32970700<br>H -1.05526800 -1.59611200 -1.29932900<br>H -0.36301300 -1.88439300 0.31239600<br>H -0.65659800 1.48136400 0.97763200<br>H -0.69684400 2.01000600 -0.72171700<br>H -3.04387700 1.29326800 0.52094700<br>H -2.74917300 0.77375200 -1.14028900<br>H -2.19592900 -0.81258800 1.41644900<br>H -3.08516700 -1.36955800 -0.00213500<br>B 1.43881200 0.00411300 0.04554300<br>F 1.89816100 1.23275300 -0.32234400<br>F 1.37691500 -0.19204300 1.40208000<br>F 1.94506800 -1.05295600 -0.64815700
Tetrahydropyran<br>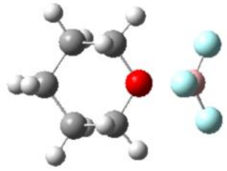	C 0.59093100 -1.21213500 -0.25938100<br>C 2.02039200 -1.25750400 0.26082000<br>C 2.79548200 0.00011800 -0.15607200<br>C 2.02023300 1.25761600 0.26099400<br>C 0.59090800 1.21194400 -0.25953100<br>H 0.55350100 -1.22011700 -1.35606900<br>H 1.99778600 -1.34046700 1.35372200<br>H 2.93302300 0.00026700 -1.24559500<br>H 1.99735500 1.34035800 1.35391100<br>H -0.02575800 -2.02373300 0.12323500<br>H 2.50134100 -2.16350800 -0.12299900<br>H 3.79496300 0.00006400 0.28898500

	H 2.50107300 2.16374400 -0.12260200<br>H -0.02607800 2.02352300 0.12258800<br>H 0.55390400 1.21961700 -1.35627200<br>O -0.06386200 -0.00005500 0.20137500<br>B -1.77616600 0.00000600 0.03332700<br>F -2.11149700 -1.14555300 0.66046800<br>F -1.94332900 -0.00000700 -1.30948900<br>F -2.11151400 1.14560700 0.66040900
Acetone<br>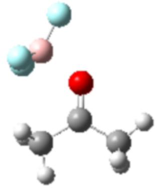	C 1.36334200 -0.10606600 0.00017800<br>O 0.29796500 -0.73084700 0.00000600<br>B -1.26461000 -0.06376500 -0.00001800<br>F -2.03940000 -1.16904200 -0.00129400<br>F -1.29615500 0.67852300 -1.14821800<br>F -1.29706800 0.67648500 1.14947600<br>C 1.42085200 1.38595600 0.00002800<br>H 0.88145700 1.76135500 0.87065400<br>H 2.44390300 1.75007500 0.00164000<br>H 0.88468200 1.76077100 -0.87288800<br>C 2.63624500 -0.88334800 -0.00003300<br>H 3.22843700 -0.60340400 -0.87458200<br>H 3.22795500 -0.60440400 0.87516500<br>H 2.44387200 -1.95174300 -0.00066100
Ethyl acetate<br>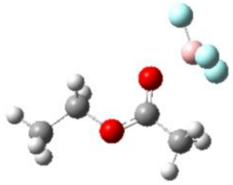	C 3.88595600 -0.57593900 -0.00006100<br>H 4.21747300 -0.03486900 -0.88543200<br>H 4.21756000 -0.03474400 0.88520100<br>H 4.36331300 -1.55597900 -0.00001400<br>C 2.38823000 -0.75645900 0.00002900<br>H 2.02866600 -1.28166900 -0.88256100<br>H 2.02876400 -1.28160600 0.88269600<br>O 1.78654300 0.58086700 0.00001600<br>C 0.48065600 0.67295300 0.00009100<br>O -0.21147600 -0.35460700 0.00007400<br>C -0.04977700 2.06492200 -0.00005200<br>H 0.76038600 2.78594400 -0.00008200<br>H -0.68687600 2.19525400 -0.87415100<br>H -0.68695900 2.19539000 0.87396000<br>B -1.89309100 -0.40865400 -0.00000100<br>F -2.24518900 0.24928800 -1.14747500<br>F -2.13261100 -1.74003500 -0.00092000<br>F -2.24528800 0.24770300 1.14835300
γ-Butyrolactone	O 0.48375900 -0.90386700 -0.06649200<br>C -0.64659300 -0.42815500 -0.00013200<br>O -1.68622200 -1.23553100 -0.06006600<br>B 1.88002200 0.04584400 -0.00549600<br>F 2.86430300 -0.87532000 -0.03898400<br>F 1.77614900 0.84207800 -1.11530200

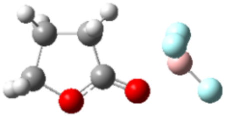	F 1.74960200 0.73033900 1.17462600<br>C -1.06448200 1.00684500 0.15855800<br>H -0.45522100 1.65206100 -0.46768300<br>H -0.86721300 1.28924000 1.19498600<br>C -2.93069300 -0.48586300 0.10023800<br>H -3.64560800 -0.91771000 -0.59296500<br>H -3.26926800 -0.64900500 1.12262800<br>C -2.55713000 0.96930200 -0.18599000<br>H -3.14984900 1.66441200 0.40307200<br>H -2.71033700 1.20031200 -1.23919800
Dimethyl carbonate<br>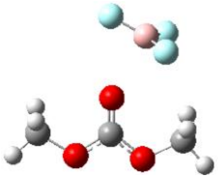	C -1.35888100 0.21539800 -0.00039100<br>O -0.27618800 -0.33194700 -0.00204400<br>O -2.52818900 -0.41649500 0.00057000<br>O -1.58026200 1.52362900 0.00061300<br>C -2.46172000 -1.85529600 -0.00015100<br>H -3.49467600 -2.18679400 0.00155400<br>H -1.94607400 -2.21012600 -0.88976200<br>H -1.94286400 -2.21090000 0.88727300<br>C -0.41226600 2.36895100 -0.00037300<br>H -0.79804100 3.38277100 0.00041600<br>H 0.18740300 2.18728000 0.88835300<br>H 0.18541200 2.18790500 -0.89057900<br>B 2.05542200 -0.36680600 0.00037900<br>F 2.09311700 -1.68538200 -0.00002100<br>F 2.17465800 0.29275800 1.14253000<br>F 2.17734100 0.29352000 -1.14103900
Nitrobenzene<br>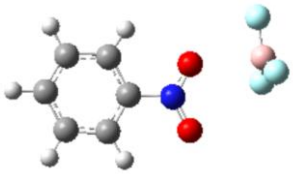	C 3.69496300 -0.85173600 0.00004200<br>C 2.34702400 -1.17893000 -0.00004000<br>C 1.41561200 -0.14906500 -0.00008900<br>C 1.78750300 1.18886000 -0.00005000<br>C 3.13976900 1.49874900 0.00003200<br>C 4.09061300 0.48261100 0.00007800<br>H 4.43593300 -1.63846700 0.00007800<br>H 2.01158600 -2.20399500 -0.00007100<br>H 1.02984100 1.95619700 -0.00008700<br>H 3.45041800 2.53386900 0.00006000<br>H 5.14300100 0.73118600 0.00014300<br>N -0.01423200 -0.49321000 -0.00018400<br>O -0.81798300 0.44018500 -0.00006200<br>O -0.32817000 -1.66842500 -0.00008200<br>B -3.23985100 0.18617700 0.00007400<br>F -3.35689100 1.50281200 0.00018700<br>F -3.29137500 -0.47210400 -1.14347800<br>F -3.29124300 -0.47228800 1.14352500
Hexamethyl-phosphoramide (HMPA)	P 0.47684800 0.00132100 -0.11683600<br>O -0.83914400 -0.18082500 -0.87627500

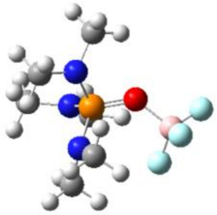	B -2.31861900 -0.21314800 -0.36134400<br>F -2.52569100 -1.45580400 0.20956400<br>F -2.42383600 0.80516200 0.59032800<br>F -3.09666400 0.01616700 -1.46474100<br>N 0.45544400 -0.23836200 1.51946800<br>N 1.52857800 -1.14126900 -0.69022100<br>N 0.99529700 1.54130400 -0.43587400<br>C 2.36334900 1.95917300 -0.16401700<br>H 2.75664700 2.51375300 -1.01921900<br>H 2.42203700 2.60884700 0.71577600<br>H 3.00827400 1.10127700 0.00536500<br>C 0.08038000 2.63283300 -0.77285000<br>H 0.01590900 3.35737100 0.04465600<br>H 0.44579800 3.14916100 -1.66334700<br>H -0.91315900 2.24834800 -0.97223700<br>C 2.73563300 -1.57891000 -0.00154600<br>H 3.63579500 -1.21704800 -0.50933500<br>H 2.74457600 -1.22073600 1.02331600<br>H 2.77747200 -2.67099900 0.01406100<br>C 1.44943700 -1.56597400 -2.08894200<br>H 1.53015100 -2.65361100 -2.14164900<br>H 0.49679100 -1.27359500 -2.51808400<br>H 2.26054500 -1.13019800 -2.68030700<br>C 0.16866400 0.85954200 2.44638300<br>H -0.89963600 0.92868600 2.65763800<br>H 0.71002800 0.68286200 3.37754400<br>H 0.49971800 1.80684400 2.03223200<br>C 0.07965700 -1.55438400 2.05609500<br>H -0.97895600 -1.57936900 2.31670300<br>H 0.26101500 -2.33233800 1.32028900<br>H 0.67830300 -1.76185400 2.94534300
Trimethylphosphine<br>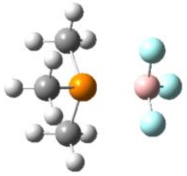	P -0.76306600 0.00001300 0.00020300<br>C -1.46895500 -1.43974800 -0.87560400<br>H -1.11601800 -1.43507700 -1.90569500<br>H -1.11550100 -2.35222300 -0.39796500<br>H -2.55833800 -1.42246000 -0.86474400<br>C -1.46949800 1.47774800 -0.80951500<br>H -1.11739000 2.36791400 -0.29054200<br>H -1.11586100 1.52034700 -1.83850400<br>H -2.55887200 1.45892500 -0.80023300<br>C -1.47124900 -0.03829200 1.68398100<br>H -1.11902600 -0.93257300 2.19574000<br>H -1.11904000 0.83179200 2.23588200<br>H -2.56058700 -0.03781600 1.66167200<br>B 1.33659000 0.00009100 0.00039200<br>F 1.68920000 -1.17028600 0.63765900<br>F 1.68909800 1.13735900 0.69507100

	F 1.68857700 0.03318100 -1.33204000
Tetrahydrothiophene<br>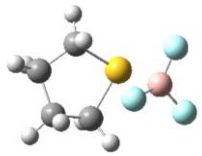	C -1.16117800 -1.30836600 -0.48173200<br>C -1.22149600 1.36290500 -0.05591000<br>C -2.46979300 0.59002200 0.37239300<br>C -2.03019800 -0.84161400 0.68564300<br>H -1.75619300 -1.63957800 -1.33010600<br>H -0.45374500 -2.08926800 -0.21639800<br>H -0.62900100 1.68644500 0.79599700<br>H -1.43926500 2.21839800 -0.68898500<br>H -2.93677200 1.06980400 1.23315800<br>H -3.20048700 0.58758000 -0.43909800<br>H -1.44500100 -0.85823700 1.60475200<br>H -2.88334000 -1.50811900 0.81786100<br>B 1.66654100 -0.01180200 0.23356000<br>F 2.27201300 1.16560700 -0.03276800<br>F 1.16154700 -0.14669900 1.48721100<br>F 2.21833000 -1.12400000 -0.30053400<br>S -0.19749500 0.17000800 -1.02828300
